# Do early life factors explain the educational differences in early labour market exit? A register-based cohort study

**DOI:** 10.1186/s12889-023-16626-3

**Published:** 2023-08-31

**Authors:** Emma Carlsson, Tomas Hemmingsson, Jonas Landberg, Bo Burström, Emelie Thern

**Affiliations:** 1 Department of Public Health Sciences, Stockholm University, SE-106 91 Stockholm, Sweden; 2 Department of Global Public Health, Karolinska Institutet, Stockholm, Sweden; 3 Institute of Environmental Medicine, Karolinska Institutet, Stockholm, Sweden; 4 Department of Clinical Neuroscience, Karolinska Institutet, Stockholm, Sweden

**Keywords:** Socioeconomic inequalities, Early retirement, Disability pension, Sickness absence, Unemployment, Older workers

## Abstract

**Background:**

Socioeconomic inequalities in labour market participation are well established. However, we do not fully know what causes these inequalities. The present study aims to examine to what extent factors in childhood and late adolescence can explain educational differences in early labour market exit among older workers.

**Methods:**

All men born in 1951–1953 who underwent conscription examination for the Swedish military in 1969–1973 (*n* = 145 551) were followed from 50 to 64 years of age regarding early labour market exit (disability pension, long-term sickness absence, long-term unemployment and early old-age retirement with and without income). Early life factors, such as cognitive ability, stress resilience, and parental socioeconomic position, were included. Cox proportional-hazards regressions were used to estimate the association between the level of education and each early labour market exit pathway, including adjustment for early life factors.

**Results:**

The lowest educated men had a higher risk of exit through disability pension (HR: 2.72), long-term sickness absence (HR: 2.29), long-term unemployment (HR: 1.45), and early old-age retirement with (HR: 1.29) and without income (HR: 1.55) compared to the highest educated men. Factors from early life explained a large part of the educational differences in disability pension, long-term sickness absence and long-term unemployment but not for early old-age retirement. Important explanatory factors were cognitive ability and stress resilience, whilst cardiorespiratory fitness had negligible impact.

**Conclusions:**

The association between education and early exit due to disability pension, long-term sickness absence and long-term unemployment was to a large part explained by factors from early life. However, this was not seen for early old-age retirement. These results indicate the importance of taking a life-course perspective when examining labour market participation in later working life.

**Supplementary Information:**

The online version contains supplementary material available at 10.1186/s12889-023-16626-3.

## Background

In Sweden, as in many European countries, pension systems are reformed by raising the retirement age to extend working life, and to adapt to population ageing and the financial challenges that follow [1, 2]. However, almost 20% of people aged 55–64 in Sweden did not participate in the labour force in 2022 [3]. To extend working life beyond normative retirement age people have to be able to work until 65 years of age and above. Those with lower education are at higher risk of exiting the labour market early compared to higher educated [4–14]. Furthermore, blue-collar workers exit the labour market almost 5 years earlier than white collar-workers, in Sweden [15]. Previously, disability pension has been the most common early exit route in Sweden but due to system changes, alternative early exit routes, such as unemployment and early old-age retirement, have increased [16]. Several factors, such as poor health and poor working conditions, have been associated with the socioeconomic inequalities seen in early exits from the labour market [6–14, 17]. However, only a few studies investigated how factors measured before labour market entry could explain socioeconomic inequalities in early labour market exits [11–14]. In this context, factors from childhood and adolescence are considered especially important since these are present during sensitive periods in the life course, according to life course theory [18, 19].

Previous research, where educational differences have not been the focus, has shown that a variety of factors measured in early life affect later labour market participation. Intelligence measured in youth is associated with both long-term sickness [13, 20], disability pension [11–14, 21, 22], and unemployment [13, 14]. Low cardiorespiratory fitness [23] and high body mass index (BMI) [12–14, 22, 23], as well as psychiatric and musculoskeletal diagnoses [12–14, 22] are risk factors for disability pension. Psychiatric diagnoses [13, 14, 24] and stress resilience [25] are associated with unemployment. Several of these early life factors tend to be unequally distributed across groups with different levels of education [7, 21, 26].

However, only a few studies examined the association between education and early exit from the labour market including early life factors. These demonstrate that early life factors influence the association, although it cannot entirely be explained by these factors [11–14]. Especially cognitive ability was shown to be an important explanatory factor. However, to our knowledge, the importance of other early life factors, such as cardiorespiratory fitness, muscle strength and stress resilience, has not been examined. Furthermore, several of these studies evaluated disability pension as the only exit route [11, 12]. Since regulations change over time, it is important to have a broader perspective and examine more than one exit route. Moreover, labour market exit was measured at different ages, but only rarely up until 65 years of age [14], which is the normative retirement age in Sweden.

The present study aims to investigate to what extent factors in childhood and late adolescence contribute to explain the educational inequalities in early labour market exit among older workers. This study extends previous research by: *(1)* following men from 50 years of age up until the Swedish normative retirement age *(2)* examining five different exit routes (disability pension, long-term sickness absence, long-term unemployment, old-age retirement with and without income) and *(3)* including additional early life factors.

## Methods

### Study population

**Fig. 1:**
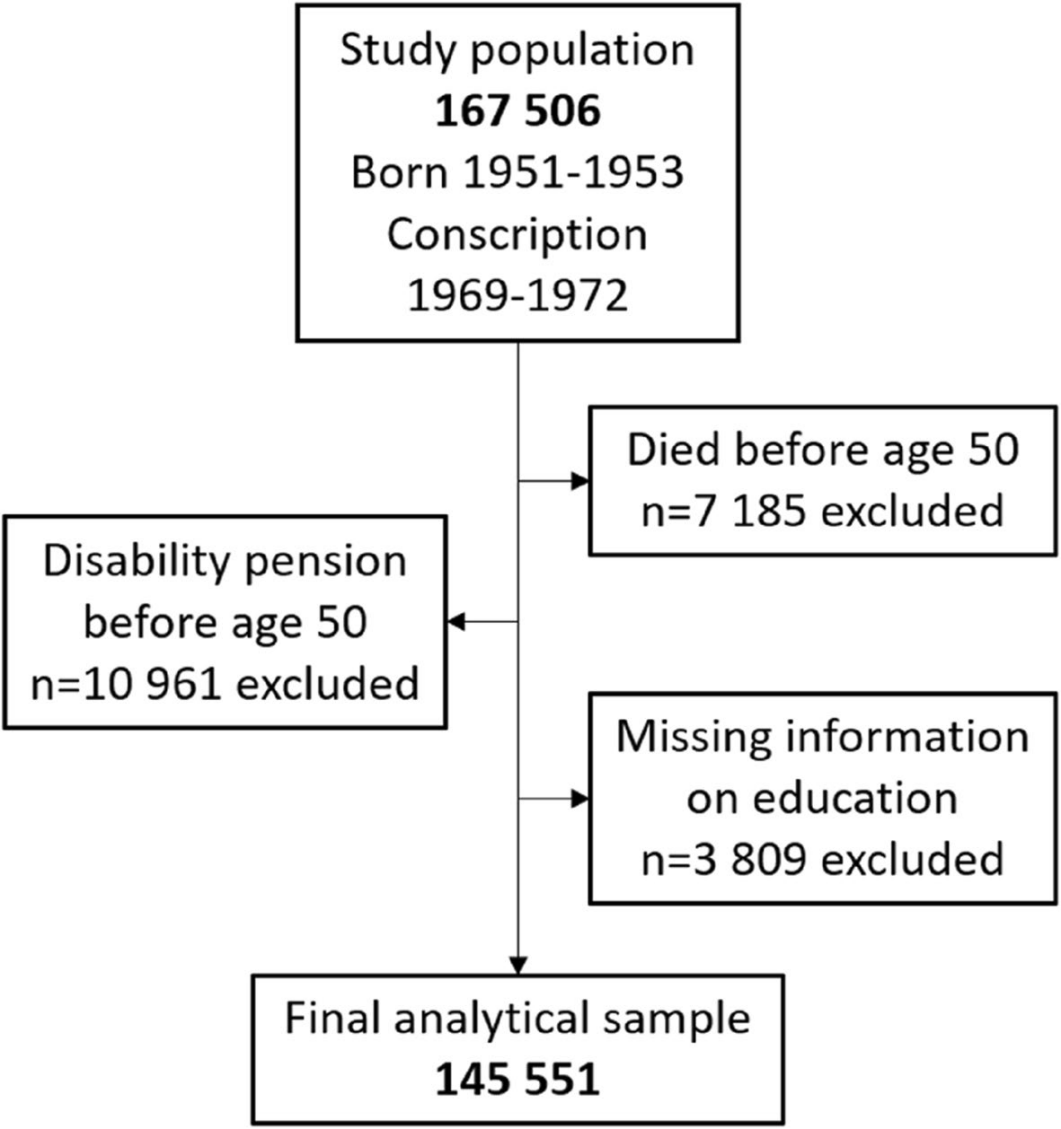
Flow chart describing the selection process of the participants

This cohort study included all men born between 1951 and 1953 who underwent compulsory conscription examination for military service at age 18 to 20 years in Sweden (*n* = 167 506). Only 2–3% of all men were exempted from the examination, due to severe disease or disabilities, since military service was obligatory by law for all males aged 18–20 years at this time [27]. All men that underwent the conscription examination are included in the study population, however, all of them did not enter the military service after the examination, since some refused, were diseased or participated in civilian service instead. The study population included all men alive at age 50. Subjects who received disability pension the year before the follow-up started were excluded (*n* = 10 961) as well as individuals with missing data on education (*n* = 3 809). Excluded individuals had lower educational levels, lower parental socioeconomic position (SEP) during childhood and worse health in late adolescence (Supplementary Table 1 in Additional file 1). The final analytical sample consisted of 145 551 (87%) men (see Fig. 1).

### Level of education (exposure)

Information on the level of education was collected from the Longitudinal Register of Education and Labour Market Statistics (LISA) when the men were 49 years old. The LISA-register was established in 1990, is updated yearly by Statistics Sweden and contains information about education, income and employment for all Swedish citizens [28]. Subjects were divided into five categories based on number of years in education: = 9 years (primary school), 10–11 years (1–2 years of upper secondary), 12 years (3 years of upper secondary), 13–14 years (1–2 years of university education) and = 15 years (at least 3 years of university), where the last group was set as the reference category, in line with previous research [12–14].

### Early labour market exit (outcome)

Early labour market exit was defined using five different exit routes: disability pension, long-term sickness absence, long-term unemployment, early old-age retirement with income and early old-age retirement without income. Information on exit was collected from the LISA-register from 1 January the year the subjects turned 50 years old up until 31 December the year the subjects turned 64 years old. In Sweden, individuals aged 30 to 64 with a medically verified injury or disease, and at least 25% reduced work capacity, can be granted disability pension from the Swedish Social Insurance Agency [28]. In this study, disability pension was defined as all subjects with full- or part-time disability pension, as in previous research [13, 14]. Previous research defines long-term sickness absence as receiving sickness benefits for 90 annual days or more from the Swedish Social Insurance Agency [13, 14, 29]. We extended the definition to those receiving benefits for a minimum of 90 days in one year and a minimum of 90 days the following year. Long-term unemployment was defined as being registered as full-time unemployed at the Swedish Public Employment Service for a minimum of 180 days in one year and a minimum of 180 days the following year. This definition was extended from definitions in previous research [13, 14, 29]. These two outcomes were created to be stricter compared to earlier studies, to be able to capture individuals that are further away from being active in the labour market. Early old-age retirement, defined as receiving any old-age pension annually, can in Sweden be granted after the individual turn 61 years of age, and while still having a paid job and an income. When analysing early old-age retirement, men who died or received disability pension between the ages 50 to 61 were excluded from these analyses (*n* = 14 783). Early old-age retirement with income was defined as receiving any old-age pension and having an income above one Price Base Amount (PBA) (approximately 4500 euro per year) the following year, whereas early old-age retirement without income was defined as receiving any old-age pension and having an income below one PBA the following year, as in previous research [14].

### Potential explanatory factors

**Fig. 2:**
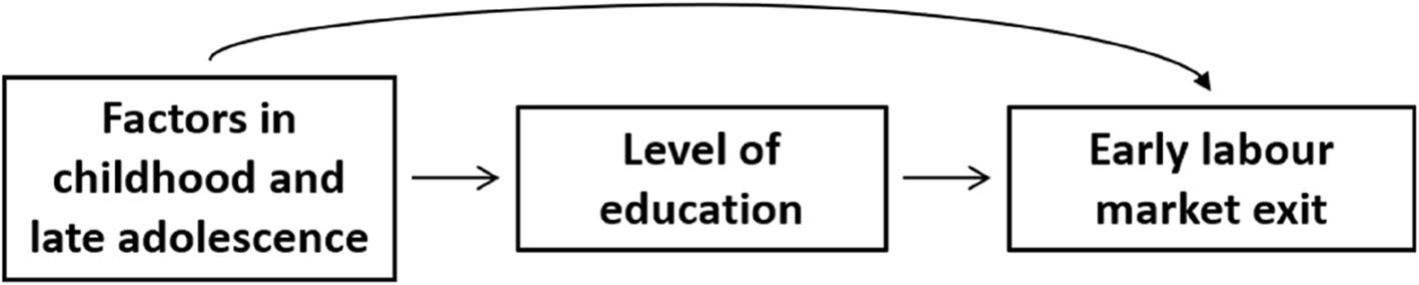
Hypothesized model of the confounding effects of factors in childhood and late adolescence on the relation between educational level and early labour market exit

Factors measured during childhood and late adolescence associated with education and labour market exit according to previous research were included as potential explanatory factors [11–14]. These factors may act as confounders in the relationship between education and labour market exit (see Fig. 2).

#### Factors in childhood

Information on childhood SEP was obtained from the National Population and Housing Census of 1960 and 1970. Childhood SEP was measured through parental occupational class, parental educational level, and crowded housing during childhood. Parental occupational class and crowded housing were obtained the year 1960 when the participants were 7–9 years old. Parental educational level was obtained year 1970 when the participants were 17–19 years old, since this information did not exist in the register year 1960. For occupational class and education, the value from the parent with the highest level in each variable was used, respectively. Occupations were classified into seven groups according to the Swedish socioeconomic classification of occupations: unskilled workers, skilled workers, low-level non-manual employees, intermediate non-manual employees, high-level non-manual employees, self-employed or farmers, and those not classified [30]. Parental level of education was classified similarly to the participants’ educational level, with at least three years of university education as the highest level. Crowded housing was defined as living in a household with more than two individuals per room, excluding the kitchen, according to Statistics Sweden [31].

#### Factors in late adolescence

At the mandatory conscription examination, during late adolescence, all men went through extensive psychological and physiological tests, described in detail elsewhere [27]. This study includes results from seven of these tests: BMI, cognitive ability, stress resilience, cardiorespiratory fitness, muscle strength, and psychiatric and musculoskeletal diagnoses. Measurements of body height and weight were used to calculate BMI (weight in kg/height in m²) and then dichotomised into BMI 25 and above (classified as overweight or obese), or below BMI 25. Cognitive ability was measured through scores from an intelligence test covering synonyms, induction, spatial capacity, and technical abilities. The scores were converted to a nine-grade scale (1-9), with higher values indicating higher cognitive ability. Stress resilience was rated on a nine-level scale by a psychologist after an interview including areas such as emotional stability, social maturity and coping with stress. Cardiorespiratory fitness was measured with a stationary cycle ergometer through maximum work rate in watts divided by body weight and converted to a nine-grade scale. Muscle strength was measured by three isometric tests (knee extension, elbow flexion and handgrip) giving a weighted sum transformed into a nine-level scale. For all factors using a nine-level scale, a higher number indicates better functioning and for this study, these were categorised into three groups, low (1-3), medium (4-6) and high (7-9). All conscripts were examined by a physician, and psychiatric and musculoskeletal diagnoses were obtained according to the International Classification of Disease version 8 (ICD-8); 290–315 and 710–738, respectively.

### Statistical analysis

Pearson’s chi-square tests were used to test for differences in baseline characteristics by educational level. Cox proportional-hazards regressions were used to estimate the association between the level of education and each early labour market exit pathway, using age as the timescale. Hazard ratios (HRs) are presented with 95% confidence intervals (CIs). The proportional hazards assumption was tested using Schoenfeld residuals and the global test was significant (*p*-value < 0.05) for disability pension, long-term unemployment, early old-age retirement without income (but not for long-term sickness absence and early old-age retirement with income). However, the non-proportionality was considered small when examined further by plotting the log cumulative hazard function. Adjustments were made for all explanatory factors, individually at first and then grouped, first by all childhood variables, then all variables from late adolescence and ultimately the full model including all explanatory factors. Missing values on the explanatory factors were coded as separate categories since similar results were obtained when analysing complete cases (Supplementary Tables 2 and 3 in Additional file 1). For the explanatory variables parental educational level and crowded housing, there were missing values due to lack of data in the registers (see Table 1). For the explanatory variables from late adolescence, there were missing values due to internal missing in the data [27]. To analyse to what extent the explanatory factors could explain the association between education and early labour market exit the percentage of the hazard ratio reduction after adjusting for the factor was calculated as ((HRcrude-HRadjusted)/(HRcrude-1)) *100. For example, if the hazard ratio attenuates from HR 2.0 in the crude model to HR 1.5 after adjusting for the explanatory factors, the interpretation is that the explanatory factors explain 50% of the increased risk.

**Table 1:** Baseline characteristics of study population, stratified by years of education

Years of education	≥ 15 n(%)	13-14 n(%)	12 n(%)	10-11 n(%)	≤ 9 n(%)	*p*-value
**Total**	**24 439 (16.8)**	**20 677 (14.2)**	**21 136 (14.5)**	**45 668 (31.4)**	**33 631 (23.1)**	
*Childhood variables*						
Parental education						
≤ 9	8703 (35.6)	10 428 (50.4)	11 718 (55.4)	29 935 (65.6)	25 153 (74.8)	<0.001
10-11	4 703 (19.2)	4 268 (20.6)	4 166 (19.7)	8 097 (17.7)	3880 (11.5)	
12	3 074 (12.6)	2 282 (11.0)	1 893 (9.0)	2 586 (5.6)	1 109 (3.3)	
13-14	1 714 (7.0)	963 (4.7)	668 (3.1)	774 (1.7)	316 (1.0)	
≥ 15	4 364 (17.9)	1 260 (6.1)	1 026 (4.9)	592 (1.3)	239 (0.7)	
Missing	1 881 (7.7)	1 476 (7.1)	1 665 (7.9)	3 684 (8.1)	2 934 (8.7)	
Parental occupation						
Unskilled worker	3 560 (14.6)	4 546 (22.0)	5 290 (25.0)	14 736 (32.3)	12 107 (36.0)	<0.001
Skilled worker	3 392 (13.9)	4 281 (20.7)	4 622 (21.9)	11 733 (25.7)	8 426 (25.0)	
Low-level non-manual employee	3 663 (15.0)	2 824 (13.7)	2 856 (13.5)	4 410 (9.7)	2 425 (7.2)	
Intermediate non-manual employee	7 085 (29.0)	4 468 (21.6)	3 643 (17.2)	4 969 (10.9)	2 410 (7.2)	
High-level non-manual employee	3 437 (14.0)	1 206 (5.8)	1 137 (5.4)	978 (2.1)	566 (1.7)	
Farmer	1 318 (5.4)	1 761 (8.5)	1 792 (8.5)	4 489 (9.8)	4 592 (13.7)	
Not classified	1 984 (8.1)	1 591 (7.7)	1 796 (8.5)	4 353 (9.5)	3 105 (9.2)	
Crowded housing	2 194 (9.0)	2 952 (14.3)	3 414 (16.1)	10 268 (22.5)	8 608 (25.6)	<0.001
Missing	1 386 (5.7)	970 (4.7)	1 117 (5.3)	2 452 (5.4)	1 716 (5.1)	
*Late adolescence variables*						
Cognitive ability						
High (7-9)	14 307 (58.5)	9 026 (43.7)	7 154 (33.8)	6 657 (14.6)	2 763 (8.2)	<0.001
Medium (4-6)	7 994 (32.7)	9 538 (46.1)	10 799 (51.1)	25 965 (56.9)	16 838 (50.1)	
Low (1-3)	533 (2.2)	989 (4.8)	1 915 (9.1)	10 117 (22.1)	11 646 (34.6)	
Missing	1 605 (6.6)	1 124 (5.4)	1 268 (6.0)	2 929 (6.4)	2 384 (7.1)	
Stress resilience						
High (7-9)	8 356 (34.2)	6 323 (30.6)	5 484 (26.0)	7 883 (17.3)	4 718 (14.0)	<0.001
Medium (4-6)	11 193 (45.8)	10 533 (50.9)	11 223 (53.1)	24 855 (54.4)	17 396 (51.7)	
Low (1-3)	3 197 (13.1)	2 632 (12.7)	3 087 (14.6)	9 822 (21.5)	8 991 (26.7)	
Missing	1 693 (6.9)	1 189 (5.8)	1 342 (6.3)	3 108 (6.8)	2 526 (7.5)	
BMI ≥ 25	817 (3.3)	1 004 (4.9)	1 087 (5.1)	2 998 (6.5)	2 683 (8.0)	<0.001
Missing	2 323 (9.5)	1 753 (8.5)	1 843 (8.7)	4 544 (10.0)	3 356 (10.0)	
Muscle strength						
High (7-9)	4 008 (16.4)	4 311 (20.9)	4 270 (20.2)	9 874 (21.6)	7 888 (23.5)	<0.001
Medium (4-6)	15 003 (61.4)	12 800 (61.9)	12 991 (61.5)	27 338 (59.9)	19 620 (58.3)	
Low (1-3)	3 856 (15.8)	2 467 (11.9)	2 627 (12.4)	5 540 (12.1)	3 747 (11.1)	
Missing	1 572 (6.4)	1 099 (5.3)	1 248 (5.9)	2 916 (6.4)	2 376 (7.1)	
Cardiorespiratory fitness						
High (7-9)	11 549 (47.2)	9 417 (45.5)	8 730 (41.3)	15 743 (34.5)	9 786 (29.1)	<0.001
Medium (4-6)	10 454 (42.8)	9 360 (45.3)	10 158 (48.1)	24 207 (53.0)	18 975 (56.4)	
Low (1-3)	854 (3.5)	794 (3.8)	994 (4.7)	2 773 (6.1)	2 467 (7.3)	
Missing	1 582 (6.5)	1 106 (5.4)	1 254 (5.9)	2 945 (6.4)	2 403 (7.1)	
Psychiatric diagnoses	2 250 (9.2)	1 826 (8.8)	2 104 (10.0)	6 583 (14.4)	5 872 (17.5)	<0.001
Musculoskeletal diagnoses	3 658 (15.0)	3 237 (15.7)	3 290 (15.6)	7 571 (16.6)	5 654 (16.8)	<0.001
*Outcome*						
Disability pension	1 213 (5.0)	1 409 (6.8)	1 796 (8.5)	5 545 (12.1)	4 358 (13.0)	<0.001
Long-term sickness absence	1 807 (7.4)	2 026 (9.8)	2 520 (11.9)	7 258 (15.9)	5 453 (16.2)	<0.001
Long-term unemployment	1 105 (4.5)	1 202 (5.8)	1 374 (6.5)	3 573 (7.8)	2 185 (6.5)	<0.001
Early old-age retirement without income	4 958 (21.5)	5 403 (28.1)	5 813 (30.2)	12 228 (30.5)	9 281 (31.8)	<0.001
Early old-age retirement with income	3 391 (14.7)	3 306 (17.2)	3 157 (16.4)	7 232 (18.1)	5 367 (18.4)	<0.001

For all outcomes, each model was run separately, consequently, an individual could be observed in several different outcomes. For the outcomes disability pension, long-term sickness absence and long-term unemployment person-time were counted from age 50 years (1 of January 2001 at the earliest) until the outcome of interest, emigration, death or until age 64 years (31 December 2017 at the latest), whichever came first. Since early old-age retirement only can be granted from age 61 in Sweden, person-time for the outcome early old-age retirement with and without income was counted from age 61 (1 January 2012 at the earliest) until the outcome of interest, emigration, death or until age 64 years (31 December 2017 at the latest), whichever came first. Information about the various outcomes came from registers that are updated annually and there were no exact dates on when these were received. Consequently, the day and the month of the outcome were set to the middle of the year, the 2nd of July, as in previous research [13, 14]. All analyses were performed using Stata Statistical Software: Release 17.

## Results

A description of the study population, stratified by level of education, can be found in Table 1. Participants with lower levels of education also had lower SEP in childhood, lower cognitive ability, lower stress resilience, lower cardiorespiratory fitness, higher BMI, and were more often diagnosed with a psychiatric or musculoskeletal diagnosis compared to those with higher levels of education. Muscle strength was the only variable with the opposite direction, where those with lower education had a higher proportion of men in the high category, indicating better muscle strength among lower educated compared to higher educated.

During the follow-up, 14 321 (9.84%) men exited the labour market due to disability pension, 19 064 (13.1%) exited through long-term sickness absence, 9 439 (6.49%) men exited through long-term unemployment, 37 683 (28.82%) through early old-age retirement without income and 22 453 (17.17%) men exited through early old-age retirement with income.

Almost all early life factors included in the analysis were positively associated with each outcome, when analysed separately (Table 2), with some exceptions, for example, muscle strength, and especially for early old-age retirement with and without income.

**Table 2:** Unadjusted HRs and 95% CIs on the association between each potential explanatory factor and each outcome separately

	Disability pension	Long-term sickness absence	Long-term unemployment	Old-age retirement without income	Old-age retirement with income
*Childhood variables*					
Parental education					
≤ 9	2.04 (1.84-2.26)	1.87 (1.72-2.04)	1.18 (1.06-1.30)	1.30 (1.23-1.36)	1.12 (1.06-1.19)
10-11	1.76 (1.58-1.96)	1.66 (1.52-1.82)	1.20 (1.07-1.33)	1.25 (1.19-1.32)	1.09 (1.02-1.17)
12	1.56 (1.38-1.76)	1.53 (1.38-1.69)	1.26 (1.11-1.42)	1.24 (1.17-1.32)	1.01 (0.93-1.09)
13-14	1.34 (1.15-1.56)	1.26 (1.11-1.43)	1.19 (1.02-1.39)	1.11 (1.03-1.20)	0.99 (0.90-1.09)
≥ 15	1.00	1.00	1.00	1.00	1.00
Parental occupation					
Unskilled worker	1.81 (1.64-2.00)	1.62 (1.49-1.75)	1.25 (1.12-1.39)	1.22 (1.16-1.29)	1.08 (1.01-1.15)
Skilled worker	1.75 (1.59-1.94)	1.57 (1.45-1.70)	1.22 (1.09-1.36)	1.21 (1.15-1.28)	1.05 (0.99-1.12)
Low-level non-manual employee	1.39 (1.25-1.55)	1.28 (1.17-1.40)	1.20 (1.07-1.35)	1.22 (1.15-1.29)	1.00 (0.93-1.07)
Intermediate non-manual employee	1.30 (1.17-1.44)	1.15 (1.06-1.26)	1.24 (1.11-1.38)	1.13 (1.07-1.20)	0.98 (0.91-1.04)
High-level non-manual employee	1.00	1.00	1.00	1.00	1.00
Farmer	1.28 (1.15-1.43)	1.17 (1.07-1.28)	0.76 (0.67-0.87)	1.17 (1.11-1.24)	0.84 (0.78-0.91)
Not classified	2.78 (2.51-3.08)	2.12 (1.95-2.31)	2.00 (1.79-2.25)	1.00 (0.94-1.06)	0.87 (0.81-0.94)
Crowded housing	1.32 (1.27-1.37)	1.23 (1.19-1.28)	1.21 (1.15-1.28)	1.05 (1.02-1.08)	1.08 (1.04-1.11)
*Late adolescence variables*					
Cognitive ability					
High (7-9)	1.00	1.00	1.00	1.00	1.00
Medium (4-6)	1.65 (1.57-1.73)	1.54 (1.49-1.61)	1.26 (1.20-1.33)	1.11 (1.09-1.14)	1.12 (1.09-1.16)
Low (1-3)	2.67 (2.53-2.81)	2.16 (2.07-2.26)	1.73 (1.62-1.84)	1.18 (1.14-1.22)	1.17 (1.12-1.21)
Stress resilience					
High (7-9)	1.00	1.00	1.00	1.00	1.00
Medium (4-6)	1.37 (1.31-1.44)	1.23 (1.18-1.28)	1.28 (1.21-1.36)	1.03 (1.01-1.06)	0.98 (0.95-1.01)
Low (1-3)	2.38 (2.25-2.51)	1.74 (1.66-1.82)	1.90 (1.78-2.02)	1.05 (1.02-1.09)	0.94 (0.91-0.98)
BMI ≥ 25	1.47 (1.38-1.56)	1.47 (1.40-1.55)	1.08 (0.99-1.17)	1.06 (1.02-1.11)	1.05 (1.00-1.11)
Muscle strength					
High (7-9)	1.00	1.00	1.00	1.00	1.00
Medium (4-6)	0.95 (0.91-0.99)	0.90 (0.87-0.94)	1.06 (1.00-1.12)	0.94 (0.92-0.96)	0.98 (0.95-1.01)
Low (1-3)	1.00 (0.95-1.06)	0.86 (0.82-0.91)	1.26 (1.18-1.36)	0.88 (0.85-0.91)	0.89 (0.85-0.93)
Cardiorespiratory fitness					
High (7-9)	1.00	1.00	1.00	1.00	1.00
Medium (4-6)	1.34 (1.29-1.40)	1.20 (1.16-1.24)	1.25 (1.19-1.31)	1.06 (1.04-1.08)	0.98 (0.96-1.01)
Low (1-3)	1.64 (1.53-1.76)	1.34 (1.26-1.43)	1.53 (1.41-1.67)	1.04 (0.99-1.09)	0.93 (0.88-0.99)
Psychiatric diagnoses	1.90 (1.83-1.98)	1.53 (1.48-1.59)	1.49 (1.42-1.57)	1.05 (1.02-1.08)	0.97 (0.93-1.01)
Musculoskeletal diagnoses	1.16 (1.11-1.21)	1.21 (1.16-1.25)	0.97 (0.92-1.03)	1.03 (1.01-1.06)	1.04 (1.01-1.08)

### Disability pension

The association between level of education and disability pension was graded, and those with less than 12 years of education had a 2.5-fold rate of disability pension compared to those with more than 15 years of education, in the crude model (Table 3). The hazard ratios attenuated by 18–47% when including all early life factors, at the most for the group with the shortest education. The largest proportion of this attenuation was due to cognitive ability, which by itself attenuated the ratio by up to 35%. Stress resilience and factors from childhood explained parts of the reduction.

**Table 3:** Crude and adjusted hazard ratios (HRs) with 95% confidence intervals (CIs) for the association between level of education (in years) and early exit through disability pension, long-term sickness absence, and long-term unemployment, and percentage of HR reduction (%Δ) by each potential explanatory factor

Years of education	≥ 15	13-14	%∆	12	%∆	10-11	%∆	≤ 9	%∆
**Disability pension (14 321 events)**									
Crude	1.00	1.38 (1.28-1.49)		1.74 (1.62-1.87)		2.54 (2.39-2.71)		2.72 (2.56-2.90)	
*Childhood variables*									
Parental education	1.00	1.34 (1.24-1.45)	10	1.68 (1.56-1.81)	8	2.42 (2.27-2.58)	8	2.57 (2.40-2.75)	9
Parental occupation	1.00	1.36 (1.26-1.47)	5	1.70 (1.58-1.84)	5	2.45 (2.30-2.61)	6	2.64 (2.47-2.82)	5
Crowded housing	1.00	1.38 (1.28-1.49)	0	1.73 (1.61-1.86)	2	2.50 (2.35-2.66)	3	2.67 (2.50-2.85)	3
Adjusted for all childhood variables	1.00	1.35 (1.25-1.45)	9	1.68 (1.56-1.80)	9	2.39 (2.24-2.55)	10	2.57 (2.40-2.74)	9
*Late adolescence variables*									
Cognitive ability	1.00	1.33 (1.24-1.44)	12	1.61 (1.49-1.73)	18	2.12 (1.99-2.26)	27	2.12 (1.98-2.27)	35
Stress resilience	1.00	1.40 (1.29-1.51)	-4	1.72 (1.60-1.85)	3	2.38 (2.24-2.53)	11	2.45 (2.30-2.61)	16
BMI ≥ 25	1.00	1.38 (1.28-1.50)	-1	1.75 (1.62-1.88)	0	2.51 (2.36-2.67)	2	2.68 (2.51-2.85)	3
Muscle strength	1.00	1.40 (1.30-1.51)	-6	1.76 (1.64-1.89)	-2	2.55 (2.40-2.72)	-1	2.71 (2.54-2.89)	1
Cardiorespiratory fitness	1.00	1.39 (1.29-1.50)	-3	1.73 (1.61-1.86)	1	2.48 (2.33-2.64)	4	2.60 (2.44-2.77)	7
Psychiatric diagnoses	1.00	1.38 (1.28-1.50)	-1	1.74 (1.61-1.87)	1	2.46 (2.31-2.62)	5	2.58 (2.42-2.75)	8
Musculoskeletal diagnoses	1.00	1.38 (1.28-1.49)	0	1.74 (1.62-1.87)	0	2.54 (2.38-2.70)	0	2.72 (2.55-2.89)	0
Adjusted for all late adolescence variables	1.00	1.33 (1.23-1.44)	12	1.59 (1.47-1.71)	21	2.01 (1.88-2.14)	35	1.95 (1.82-2.08)	45
Full model	1.00	1.31 (1.21-1.42)	18	1.56 (1.44-1.68)	25	1.96 (1.83-2.10)	38	1.92 (1.79-2.06)	47
**Long-term sickness absence (19 064 events)**									
Crude	1.00	1.33 (1.25-1.42)		1.65 (1.55-1.75)		2.25 (2.13-2.37)		2.29 (2.18-2.42)	
*Childhood variables*									
Parental education	1.00	1.30 (1.22-1.38)	11	1.59 (1.50-1.69)	9	2.15 (2.03-2.26)	8	2.18 (2.06-2.30)	9
Parental occupation	1.00	1.32 (1.23-1.40)	5	1.61 (1.52-1.71)	6	2.16 (2.05-2.28)	7	2.22 (2.10-2.34)	6
Crowded housing	1.00	1.33 (1.25-1.42)	0	1.64 (1.54-1.74)	1	2.22 (2.11-2.34)	2	2.26 (2.14-2.39)	3
Adjusted for all childhood variables	1.00	1.30 (1.22-1.39)	10	1.59 (1.49-1.69)	10	2.12 (2.01-2.24)	10	2.17 (2.05-2.29)	10
*Late adolescence variables*									
Cognitive ability	1.00	1.29 (1.21-1.37)	13	1.54 (1.45-1.63)	17	1.94 (1.83-2.04)	25	1.88 (1.78-1.99)	32
Stress resilience	1.00	1.34 (1.26-1.43)	-2	1.64 (1.54-1.74)	2	2.16 (2.05-2.27)	7	2.15 (2.04-2.27)	11
BMI ≥ 25	1.00	1.33 (1.25-1.42)	0	1.65 (1.55-1.75)	0	2.22 (2.11-2.33)	2	2.25 (2.13-2.37)	3
Muscle strength	1.00	1.34 (1.26-1.43)	-1	1.65 (1.55-1.75)	0	2.24 (2.12-2.35)	1	2.27 (2.15-2.39)	2
Cardiorespiratory fitness	1.00	1.34 (1.26-1.43)	-2	1.64 (1.55-1.75)	1	2.21 (2.10-2.33)	3	2.23 (2.12-2.37)	5
Psychiatric diagnoses	1.00	1.34 (1.25-1.42)	-1	1.64 (1.55-1.75)	1	2.20 (2.09-2.32)	3	2.22 (2.11-2.34)	6
Musculoskeletal diagnoses	1.00	1.33 (1.25-1.42)	0	1.65 (1.55-1.75)	0	2.24 (2.13-2.36)	0	2.29 (2.17-2.41)	1
Adjusted for all late adolescence variables	1.00	1.28 (1.20-1.36)	17	1.51 (1.42-1.61)	21	1.85 (1.75-1.96)	32	1.76 (1.66-1.87)	41
Full model	1.00	1.26 (1.18-1.34)	23	1.48 (1.39-1.58)	26	1.81 (1.71-1.91)	35	1.74 (1.64-1.84)	43
**Long-term unemployment (9 439 events)**									
Crude	1.00	1.29 (1.18-1.39)		1.45 (1.34-1.57)		1.76 (1.65-1.88)		1.45 (1.35-1.56)	
*Childhood variables*									
Parental education	1.00	1.31 (1.20-1.42)	-8	1.48 (1.36-1.60)	-7	1.82 (1.69-1.95)	-8	1.50 (1.39-1.62)	-12
Parental occupation	1.00	1.31 (1.21-1.43)	-10	1.48 (1.37-1.60)	-7	1.82 (1.70-1.95)	-7	1.53 (1.42-1.65)	-17
Crowded housing	1.00	1.29 (1.19-1.40)	-1	1.44 (1.33-1.56)	2	1.73 (1.62-1.85)	4	1.42 (1.32-1.53)	6
Adjusted for all childhood variables	1.00	1.32 (1.22-1.44)	-13	1.49 (1.37-1.61)	-9	1.83 (1.70-1.96)	-9	1.54 (1.43-1.66)	-20
*Late adolescence variables*									
Cognitive ability	1.00	1.26 (1.16-1.37)	8	1.37 (1.27-1.49)	16	1.56 (1.45-1.67)	27	1.22 (1.13-1.32)	52
Stress resilience	1.00	1.29 (1.19-1.40)	-3	1.43 (1.32-1.54)	5	1.66 (1.55-1.77)	14	1.32 (1.23-1.42)	29
BMI ≥ 25	1.00	1.29 (1.19-1.40)	-3	1.45 (1.34-1.57)	-1	1.75 (1.64-1.88)	1	1.44 (1.34-1.55)	2
Muscle strength	1.00	1.31 (1.21-1.42)	-9	1.47 (1.36-1.59)	-4	1.78 (1.66-1.90)	-2	1.46 (1.36-1.57)	-2
Cardiorespiratory fitness	1.00	1.29 (1.19-1.40)	-3	1.44 (1.33-1.55)	3	1.71 (1.60-1.83)	6	1.38 (1.29-1.49)	15
Psychiatric diagnoses	1.00	1.29 (1.19-1.40)	-1	1.44 (1.33-1.56)	1	1.72 (1.61-1.84)	5	1.40 (1.30-1.51)	11
Musculoskeletal diagnoses	1.00	1.29 (1.18-1.39)	0	1.45 (1.34-1.57)	0	1.76 (1.65-1.88)	0	1.45 (1.35-1.56)	0
Adjusted for all late adolescence variables	1.00	1.27 (1.17-1.38)	5	1.37 (1.26-1.48)	17	1.51 (1.40-1.62)	33	1.16 (1.07-1.25)	65
Full model	1.00	1.31 (1.20-1.42)	-7	1.42 (1.31-1.54)	7	1.59 (1.47-1.71)	23	1.25 (1.16-1.36)	44

### Long-term sickness absence

A graded relationship was seen between the level of education and long-term sickness absence, where those in the two groups with the shortest education had a 2.3-fold rate of sickness absence compared to those with the longest education, shown in the crude model in Table 3. In the fully adjusted model, the hazard ratio attenuated by 23 to 43 per cent, with the highest attenuation for the group with 9 years of education or shorter. Cognitive ability was the variable that explained most of the attenuation, followed by stress resilience and parental education.

### Long-term unemployment

Exit through long-term unemployment followed the same pattern as long-term sickness absence except for the group with the lowest level of education which had a lower risk of unemployment compared to the group with 10 to 11 years of education (Table 3). Factors from childhood did not explain the association, however, it was to a large part explained by factors from late adolescence. Cognitive ability was the variable that by itself explained most of the attenuation, followed by stress resilience.

### Early old-age retirement

Table 4 shows the association between education and early old-aged retirement, with and without income. Men with the lowest level of education had a higher rate of both early old-age retirement with and without income, 1.29 and 1.55, respectively, compared to men with the highest education, in the crude models. Factors from childhood and late adolescence were negligible for old-age retirement without income, explaining 3–5% of the association in the full model. For old-age retirement with income, these early factors explained 5 to 11% of the association and cognitive ability contributed the most to this rate reduction.

**Table 4:** Crude and adjusted hazard ratios (HRs) with 95% confidence intervals (CIs) for the association between level of education (years) and early exit through old-age retirement with and without income, and percentage of HR reduction (%Δ) by each potential explanatory factor

Years of education	≥ 15	13-14	%∆	12	%∆	10-11	%∆	≤ 9	%∆
**Early old-age retirement without income (37 683 events)**									
Crude	1.00	1.34 (1.29-1.39)		1.47 (1.41-1.52)		1.47 (1.43-1.52)		1.55 (1.50-1.60)	
*Childhood variables*									
Parental education	1.00	1.33 (1.27-1.38)	3	1.46 (1.40-1.51)	2	1.46 (1.41-1.51)	3	1.54 (1.49-1.60)	2
Parental occupation	1.00	1.34 (1.29-1.39)	0	1.47 (1.41-1.53)	-1	1.48 (1.43-1.54)	-2	1.57 (1.51-1.63)	-3
Crowded housing	1.00	1.33 (1.28-1.39)	1	1.46 (1.41-1.52)	1	1.47 (1.42-1.52)	0	1.55 (1.49-1.60)	1
Adjusted for all childhood variables	1.00	1.33 (1.28-1.38)	3	1.46 (1.40-1.51)	2	1.47 (1.42-1.52)	1	1.55 (1.49-1.61)	0
*Late adolescence variables*									
Cognitive ability	1.00	1.33 (1.28-1.39)	1	1.46 (1.41-1.52)	1	1.47 (1.42-1.53)	0	1.55 (1.49-1.61)	0
Stress resilience	1.00	1.33 (1.28-1.39)	1	1.47 (1.41-1.52)	0	1.48 (1.43-1.53)	-1	1.56 (1.50-1.61)	-1
BMI ≥ 25	1.00	1.33 (1.28-1.39)	1	1.46 (1.41-1.52)	1	1.47 (1.43-1.52)	0	1.55 (1.50-1.60)	0
Muscle strength	1.00	1.33 (1.28-1.38)	3	1.46 (1.40-1.51)	2	1.47 (1.42-1.52)	2	1.54 (1.49-1.60)	1
Cardiorespiratory fitness	1.00	1.33 (1.28-1.38)	1	1.46 (1.41-1.52)	1	1.47 (1.42-1.52)	1	1.55 (1.49-1.60)	1
Psychiatric diagnoses	1.00	1.34 (1.29-1.39)	0	1.47 (1.41-1.52)	0	1.47 (1.42-1.52)	0	1.55 (1.50-1.60)	0
Musculoskeletal diagnoses	1.00	1.34 (1.29-1.39)	0	1.47 (1.41-1.52)	0	1.47 (1.43-1.52)	0	1.55 (1.50-1.60)	0
Adjusted for all late adolescence variables	1.00	1.33 (1.28-1.38)	3	1.46 (1.40-1.51)	2	1.46 (1.41-1.51)	3	1.53 (1.47-1.59)	3
Full model	1.00	1.32 (1.27-1.37)	5	1.45 (1.39-1.51)	4	1.46 (1.40-1.51)	4	1.53 (1.47-1.59)	3
**Early old-age retirement with income (22 453 events)**									
Crude	1.00	1.19 (1.13-1.25)		1.13 (1.08-1.19)		1.26 (1.21-1.31)		1.29 (1.23-1.34)	
*Childhood variables*									
Parental education	1.00	1.19 (1.13-1.24)	2	1.13 (1.07-1.19)	3	1.25 (1.20-1.30)	3	1.28 (1.22-1.34)	3
Parental occupation	1.00	1.20 (1.14-1.26)	-5	1.14 (1.09-1.20)	-7	1.27 (1.22-1.33)	-6	1.31 (1.25-1.37)	-8
Crowded housing	1.00	1.18 (1.13-1.24)	3	1.13 (1.07-1.18)	4	1.25 (1.20-1.30)	3	1.27 (1.22-1.33)	4
Adjusted for all childhood variables	1.00	1.19 (1.13-1.25)	-1	1.13 (1.08-1.19)	1	1.25 (1.20-1.31)	1	1.29 (1.23-1.35)	-1
*Late adolescence variables*									
Cognitive ability	1.00	1.18 (1.12-1.23)	7	1.11 (1.06-1.17)	14	1.22 (1.17-1.28)	13	1.25 (1.19-1.31)	13
Stress resilience	1.00	1.19 (1.13-1.25)	1	1.14 (1.08-1.19)	-3	1.27 (1.22-1.33)	-6	1.31 (1.25-1.37)	-9
BMI ≥ 25	1.00	1.19 (1.13-1.24)	1	1.13 (1.08-1.19)	2	1.26 (1.21-1.31)	0	1.28 (1.23-1.34)	0
Muscle strength	1.00	1.18 (1.13-1.24)	4	1.13 (1.07-1.18)	5	1.25 (1.20-1.30)	2	1.28 (1.23-1.34)	2
Cardiorespiratory fitness	1.00	1.19 (1.13-1.25)	1	1.13 (1.08-1.19)	-1	1.27 (1.21-1.32)	-3	1.30 (1.24-1.36)	-5
Psychiatric diagnoses	1.00	1.19 (1.13-1.25)	0	1.13 (1.08-1.19)	0	1.26 (1.21-1.31)	-1	1.29 (1.24-1.35)	-2
Musculoskeletal diagnoses	1.00	1.19 (1.13-1.25)	0	1.13 (1.08-1.19)	0	1.26 (1.21-1.31)	0	1.28 (1.23-1.34)	0
Adjusted for all late adolescence variables	1.00	1.17 (1.12-1.23)	9	1.11 (1.06-1.17)	14	1.23 (1.18-1.29)	10	1.26 (1.20-1.32)	9
Full model	1.00	1.18 (1.12-1.24)	6	1.12 (1.06-1.18)	11	1.24 (1.18-1.29)	9	1.27 (1.21-1.34)	5

## Discussion

The results of this study demonstrate a graded association between the level of education and the risk of early labour market exit. This association was in large part explained by early life factors, especially for exit due to disability pension, long-term sickness absence and long-term unemployment. The two most important factors were cognitive ability and stress resilience. Factors from childhood and late adolescence could to a less extent explain educational differences in exit through early old-age retirement with and without income. The association between the level of education and the risk of early labour market exit remained after considering the early life factors.

In line with previous research, we demonstrate that low educational attainment is associated with a higher risk of early exit due to disability pension [5–7, 10–14, 32], long-term sickness absence [13, 14] and unemployment [4, 5, 7, 9, 10, 13, 14], compared to higher educational attainment. However, our study seems to include a more selected group of men compared to previous research, since the educational differences seem to decrease in our population of age 50 to 64 compared to early exits among younger men (39 to 53 years of age) [12]. Previous research examined long-term sickness absence and unemployment annually [13, 14]. Extending previous research, we defined the outcome in terms of receiving long-term sickness absence benefits and long-term unemployment benefits for two consecutive years as opposed to annually to get closer to a more permanent exit route. Still, we found educational differences in these two exit routes which suggests that inequalities are present also in more permanent exits. Educational differences were visible also in exit through both early old-age retirement routes, which were the most common exit routes in this study. Previous studies have shown conflicting results with both higher risks associated with low education compared to high education [10, 14, 33] but also comparable risks for all levels of education [5, 7, 9]. A potential explanation for these conflicting results could be the different definitions used for early old-age retirement, for example self-reported [9, 10, 33] or defined as receiving pension benefits as main income without accounting for other sources of income [5, 7].

Pension system reforms are introduced to prolong working life, due to population ageing [1]. Such changes often increase the use of alternative early exit routes, such as unemployment and old-age retirement [16, 34]. It should be noted that, in Sweden, changes in the social insurance system have been made during the follow-up time of this study [35]. In 2003, disability pension was moved from the pension system to the social insurance system and the name changed from disability pension to disability benefit. In 2008, requirements for receiving disability pension and sickness benefits were made stricter, and newly admitted disability pension benefits were reduced by 70% [36]. These changes may partly explain why early old-age retirement was the most common exit route in this study, as individuals may be forced to take this route while other routes are made unavailable.

Exit through disability pension, long-term sickness absence and long-term unemployment could be seen as involuntary exits from the labour market since these exit routes are more likely to be linked to the health of an individual [7, 8]. Early old-age retirement could be seen as a more voluntary exit, where an individual could choose to exit, with or without income. Although, the results show that less educated individuals are at higher risk of exit also through these routes. A potential explanation for this may be that they have poor health but are no longer eligible for disability pension or sickness benefits, due to system changes reducing newly admitted benefits, and are therefore forced to use their old-age retirement. This explanation is supported by previous research which suggests that poor health could be a risk factor also for early old-age retirement [9]. It could also be that less educated individuals are forced to take their old-age retirement early because of a more demanding working life. For example, high physical demands at the workplace is a risk factor for early old-age retirement [37] and are more common among lower educated [38]. It is still unclear which factors contribute to the educational differences in early old-age retirement since early life factors did not explain the association in this study.

The results of the current study strengthen and extend previous research on the importance of early factors in explaining educational differences in early labour market exit. We found, in line with previous research, that factors measured before labour market entry explained a large part of the association between education and early labour market exit through disability pension, long-term sickness absence and long-term unemployment [11–14]. However, this study extends previous research by examining additional explanatory variables to the association between education and early exits, such as stress resilience, cardiorespiratory fitness and muscle strength.

A large part of the association between the level of education and the different early labour market exit routes was explained by cognitive ability alone, which is in line with previous research [11–14]. However, when excluding cognitive ability from the fully adjusted model, the attenuation of the hazard ratios where somewhat reduced, but not fully removed (shown in Supplementary Table 4 in Additional file 1). This result suggests that other factors from early life are also important when explaining educational differences in early labour market exit. It could be discussed if cognitive ability is acting as a proxy for other factors. Previous research has, for example, shown that cardiorespiratory fitness is positively related to cognitive ability [39] and that the prevalence of factors measured at the conscription examination is distributed unequally across different levels of cognition [40]. Cognitive ability is strongly related to education [21] and to many health factors, including all-cause mortality [41]. Nevertheless, when studying cognitive ability and its association with mortality, adjusting for educational level does not attenuate the association by more than half [41]. This is seen also when examining cognitive ability and risk of disability pension, where adjustment for education attenuates the association, but it is not eliminated [22]. This indicates that educational attainment and cognitive ability are not interchangeable and do not imply the exact same thing. Moreover, compared to parental SEP, cognitive ability is not a significantly stronger predictor of attained education [42].

Stress resilience explained a large part of the association between education and early exit, through disability pension, long-term sickness absence and long-term unemployment. Low stress resilience has previously been linked to lower earnings and unemployment later in life [25]. A potential explanation for this could be that individuals with low stress resilience are more likely to not be able to reach higher education and therefore end up in low-paid jobs and unemployment. Previous research support this, as unemployment throughout working life is more common among low educated and is a risk factor for an early exit [14].

Low cardiorespiratory fitness has, by previous research, been found to be associated with disability pension due to a variety of causes [23]. It has also been found to be positively associated with parental SEP [43] and acts as a predictor of cardiovascular disease [44] and all-cause mortality [45]. However, in this study, cardiorespiratory fitness explained only a small part of the educational differences in disability pension, long-term sickness absence and long-term unemployment. A potential reason for this may be that other factors, such as fitness and lifestyle habits during adulthood, may be of greater importance.

### Strengths and limitations

Using the conscription examination, linked with information from high-quality registers, is a major strength of this study. The use of registers allowed for a long follow-up of 14 years, up until normative retirement age, and for including all men still alive in Sweden at age 49. Previous studies measured early labour market exit at different ages, but generally only up until 60 years [11–13]. The use of register data was also a strength since it reduced the risk of recall and attrition bias throughout the study.

Including several different exit routes was a strength of this study. This broader approach captures the complexity of early labour market exit more accurately compared to using only one or two exit routes. Most previous studies that examined the association between socioeconomic position and early labour market exit evaluated disability pension as the only exit route [11, 12, 17], or used solely self-reported data for exit [4, 8–10]. However, it is a limitation that exit was treated as separate routes when in reality, individuals could pass through more than one route before they exit the labour market permanently. In this study, we did not distinguish between these possible trajectories and were not able to capture the potential consequences that could follow.

The use of educational attainment as the exposure variable and as a measure of SEP could potentially be a limitation. It is a stable measure compared to occupational class or income, and may therefore be less accurate among the elderly since it does not capture potential success during working life [46]. However, due to this stability, it is not affected by personal career events such as unemployment spells or societal changes such as economic crises, which in this setting is a strength. If income or occupational class would be used as a measure of SEP, then these measures could have been negatively affected long before the individual receive sickness benefits or disability pension, or take their early old-age pension. Furthermore, participants’ educational level was collected from the registers when the individual was 49 years old. This would, for most participants, be many years after they ended their education, which reduces the risk of misclassification of the exposure.

Including only men is an obvious limitation of this study. Since military service was obligatory for men at the time, the conscription examination includes almost all young men. However, since women were not forced to go into military service there is no data for women on factors from late adolescence. Previous research suggests that educational differences in early exits from the labour market are similar for men and women [4, 32] but that they may be more pronounced for men [11]. However, both disability and sickness benefits are more common among Swedish women [47].

## Conclusions

Factors from early life explained a large part, but not all, of the association between education and early labour market exit among men 50 years and older. These results indicate the importance of considering early life factors to be able to decrease later life inequalities and increase labour force participation. In other words, measures and interventions should be targeted at childhood and adolescence to increase labour force participation in older ages and extend working life beyond normative retirement age. However, further studies are needed to explore other potentially important factors taking place in different stages of life, such as working conditions, to fully understand the inequalities seen within labour market participation. This is especially important for explaining the educational differences in early exit through old-age retirement. To raise the normative retirement age while not risking increasing social inequalities, it is important to understand the underlying causes of these inequalities, such as those seen in this study.

### Supplementary Information

**Additional file 1: **Do early life factors explain the educational differences in early labour market exit? A register-based cohort study.

## Data Availability

The data that support the findings of this study are available from Statistics Sweden under license for this study, but the data is not publicly available due to restrictions that apply to the availability of these data. Data are however available from the authors, Tomas Hemmingsson (email: tomas.hemmingsson@su.se), upon reasonable request and with permission of Statistics Sweden.

## Abbreviations

BMI: Body mass index
SEP: Socioeconomic position
LISA: Longitudinal Register of Education and Labour Market Statistics
PBA: Price Base Amount
ICD-8: International Classification of Disease version 8
HRs: Hazard ratios
CIs: Confidence intervals
