# Supplementary material for: Do early life factors explain the educational differences in early labour market exit? A register-based cohort study
Source: BMC Public Health. 2023 Aug 31;23:1680. doi: 10.1186/s12889-023-16626-3 (PMC10472566; doi:10.1186/s12889-023-16626-3)
Supplement: Supplementary file 1 — Additional file 1: Do early life factors explain the educational differences in early labour market exit? A register-based cohort study. [file 12889_2023_16626_MOESM1_ESM.docx]

**Supplementary Table 1.** Baseline characteristics of the individuals included and excluded in the study population.

|  | Included n(%) | Excluded n(%) | p-value |
| --- | --- | --- | --- |
| **Total** | 145 551 (86.9) | 21 955 (13.1) |  |
| **Parental education** |  |  |  |
| ≤ 9 | 85 937 (59.0) | 12 017 (54.7) | <0.001 |
| 10-11 | 25 114 (17.3) | 3 221 (14.7) |  |
| 12 | 10 944 (7.5) | 1 389 (6.3) |  |
| 13-14 | 4 435 (3.1) | 526 (2.4) |  |
| ≥ 15 | 7 481 (5.1) | 997 (4.6) |  |
| Missing | 11 640 (8.0) | 3 805 (17.3) |  |
| **Parental occupation** |  |  |  |
| Unskilled worker | 40 239 (27.7) | 5 724 (26.1) | <0.001 |
| Skilled worker | 32 454 (22.3) | 4 325 (19.7) |  |
| Low-level non-manual employee | 16 178 (11.1) | 2 104 (9.6) |  |
| Intermediate non-manual employee | 22 575 (15.5) | 2 618 (11.9) |  |
| High-level non-manual employee | 7 324 (5.0) | 953 (4.3) |  |
| Farmer | 13 952 (9.6) | 1 230 (5.6) |  |
| Not classified | 12 829 (8.8) | 5 001 (22.8) |  |
| **Crowded housing** | 27 436 (18.9) | 4 525 (20.6) | <0.001 |
| Missing | 7 641 (5.3) | 3 282 (15.0) |  |
| **Cognitive ability** |  |  |  |
| High (7-9) | 39 907 (27.4) | 3 514 (16.0) | <0.001 |
| Medium (4-6) | 71 134 (48.9) | 8 020 (36.5) |  |
| Low (1-3) | 25 200 (17.3) | 5 216 (23.8) |  |
| Missing | 9 310 (6.4) | 5 205 (23.7) |  |
| **Stress resilience** |  |  |  |
| High (7-9) | 32 764 (22.5) | 2 704 (12.3) | <0.001 |
| Medium (4-6) | 75 200 (51.7) | 7 534 (34.3) |  |
| Low (1-3) | 27 729 (19.0) | 6 313 (28.8) |  |
| Missing | 9 858 (6.8) | 5 404 (24.6) |  |
| **BMI ≥ 25** | 8 589 (5.9) | 1 335 (6.1) | <0.001 |
| Missing | 13 819 (9.5) | 5 889 (26.8) |  |
| **Muscle strength** |  |  |  |
| High (7-9) | 30 351 (20.9) | 3 656 (16.6) | <0.001 |
| Medium (4-6) | 87 752 (60.3) | 10 296 (46.9) |  |
| Low (1-3) | 18 237 (12.5) | 2 810 (12.8) |  |
| Missing | 9 211 (6.3) | 5 193 (23.7) |  |
| **Cardiorespiratory fitness** |  |  |  |
| High (7-9) | 55 225 (37.9) | 5 491 (25.0) | <0.001 |
| Medium (4-6) | 73 154 (50.3) | 9 787 (44.6) |  |
| Low (1-3) | 7 882 (5.4) | 1 456 (6.6) |  |
| Missing | 9 290 (6.4) | 5 221 (23.8) |  |
| **Psychiatric diagnoses** | 18 635 (12.8) | 6 442 (29.34) | <0.001 |
| **Musculoskeletal diagnoses** | 23 410 (16.1) | 3 372 (15.4) | <0.01 |
| **Years of education** |  |  |  |
| ≤ 9 | 33 631 (23.1) | 4 546 (20.7) | <0.001 |
| 10-11 | 45 668 (31.4) | 4 082 (18.6) |  |
| 12 | 21 136 (14.5) | 1 048 (4.8) |  |
| 13-14 | 20 677 (14.2) | 623 (2.8) |  |
| ≥ 15 | 24 439 (16.8) | 512 (2.3) |  |
| Missing | 0 | 11 144 (50.8) |  |

**Supplementary Table 2.** Complete case analysis excluding 21 218 individuals with missing information on potential explanatory factors. Crude and adjusted hazard ratios (HRs) with 95% confidence intervals (CIs) for the association between level of education (in years) and early exit through disability pension, long-term sickness absence, and long-term unemployment, and percentage of HR reduction (%∆) by each potential explanatory factor.

| Years of education | ≥ 15 | 13-14 | %∆ | 12 | %∆ | 10-11 | %∆ | ≤ 9 | %∆ |
| --- | --- | --- | --- | --- | --- | --- | --- | --- | --- |
| **Disability pension (11 253 events)** | |  |  |  |  |  |  |  |  |
| Crude | 1.00 | 1.41 (1.29-1.54) |  | 1.76 (1.62-1.92) |  | 2.61 (2.43-2.80) |  | 2.81 (2.61-3.02) |  |
| *Childhood variables* |  |  |  |  |  |  |  |  |  |
| Parental education | 1.00 | 1.37 (1.25-1.49) | 10 | 1.70 (1.56-1.85) | 8 | 2.49 (2.31-2.68) | 8 | 2.66 (2.47-2.87) | 8 |
| Parental occupation | 1.00 | 1.39 (1.27-1.51) | 6 | 1.73 (1.59-1.88) | 5 | 2.52 (2.35-2.71) | 6 | 2.73 (2.53-2.94) | 4 |
| Crowded housing | 1.00 | 1.40 (1.28-1.52) | 3 | 1.74 (1.61-1.89) | 3 | 2.55 (2.38-2.74) | 4 | 2.73 (2.54-2.94) | 4 |
| Adjusted for all childhood variables | 1.00 | 1.36 (1.25-1.49) | 12 | 1.69 (1.55-1.84) | 10 | 2.44 (2.27-2.63) | 11 | 2.63 (2.43-2.84) | 10 |
| *Late adolescence variables* | |  |  |  |  |  |  |  |  |
| Cognitive ability | 1.00 | 1.33 (1.22-1.45) | 20 | 1.58 (1.46-1.72) | 24 | 2.10 (1.95-2.26) | 32 | 2.11 (1.95-2.28) | 39 |
| Stress resilience | 1.00 | 1.40 (1.29-1.53) | 1 | 1.72 (1.59-1.87) | 6 | 2.41 (2.25-2.59) | 12 | 2.50 (2.32-2.69) | 17 |
| BMI ≥ 25 | 1.00 | 1.40 (1.29-1.53) | 2 | 1.75 (1.61-1.90) | 1 | 2.58 (2.41-2.77) | 2 | 2.76 (2.57-2.97) | 3 |
| Muscle strength | 1.00 | 1.41 (1.30-1.54) | -1 | 1.77 (1.63-1.92) | -1 | 2.62 (2.44-2.81) | 0 | 2.82 (2.62-3.03) | -1 |
| Cardiorespiratory fitness | 1.00 | 1.40 (1.28-1.53) | 2 | 1.74 (1.60-1.89) | 4 | 2.52 (2.35-2.71) | 5 | 2.68 (2.50-2.88) | 7 |
| Psychiatric diagnoses | 1.00 | 1.42 (1.30-1.55) | -2 | 1.76 (1.62-1.91) | 0 | 2.53 (2.36-2.72) | 5 | 2.67 (2.48-2.87) | 8 |
| Musculoskeletal diagnoses | 1.00 | 1.41 (1.29-1.53) | 0 | 1.76 (1.62-1.91) | 0 | 2.60 (2.42-2.79) | 1 | 2.79 (2.60-3.00) | 1 |
| Adjusted for all late adolescence variables | 1.00 | 1.33 (1.22-1.46) | 20 | 1.56 (1.43-1.69) | 27 | 1.97 (1.83-2.13) | 40 | 1.91 (1.77-2.07) | 49 |
| Full model | 1.00 | 1.30 (1.19-1.42) | 26 | 1.53 (1.40-1.66) | 31 | 1.92 (1.78-2.08) | 43 | 1.88 (1.73-2.04) | 51 |
| **Long-term sickness absence (15 544 events)** | | |  |  |  |  |  |  |  |
| Crude | 1.00 | 1.37 (1.27-1.47) |  | 1.68 (1.57-1.80) |  | 2.31 (2.18-2.45) |  | 2.42 (2.28-2.57) |  |
| *Childhood variables* |  |  |  |  |  |  |  |  |  |
| Parental education | 1.00 | 1.33 (1.24-1.42) | 11 | 1.62 (1.52-1.74) | 8 | 2.21 (2.08-2.34) | 8 | 2.30 (2.16-2.45) | 8 |
| Parental occupation | 1.00 | 1.34 (1.25-1.44) | 6 | 1.64 (1.53-1.76) | 6 | 2.23 (2.10-2.36) | 6 | 2.34 (2.20-2.49) | 6 |
| Crowded housing | 1.00 | 1.36 (1.26-1.46) | 2 | 1.67 (1.56-1.78) | 2 | 2.27 (2.14-2.41) | 3 | 2.37 (2.23-2.52) | 3 |
| Adjusted for all childhood variables | 1.00 | 1.32 (1.23-1.42) | 12 | 1.61 (1.50-1.72) | 10 | 2.17 (2.04-2.30) | 11 | 2.27 (2.13-2.42) | 11 |
| *Late adolescence variables* | |  |  |  |  |  |  |  |  |
| Cognitive ability | 1.00 | 1.30 (1.21-1.40) | 17 | 1.55 (1.44-1.65) | 20 | 1.96 (1.84-2.08) | 27 | 1.95 (1.83-2.08) | 33 |
| Stress resilience | 1.00 | 1.36 (1.27-1.46) | 1 | 1.66 (1.55-1.77) | 4 | 2.21 (2.08-2.34) | 8 | 2.26 (2.13-2.40) | 11 |
| BMI ≥ 25 | 1.00 | 1.36 (1.27-1.46) | 2 | 1.67 (1.56-1.79) | 2 | 2.28 (2.15-2.42) | 2 | 2.38 (2.24-2.52) | 3 |
| Muscle strength | 1.00 | 1.36 (1.27-1.46) | 1 | 1.67 (1.57-1.79) | 1 | 2.30 (2.17-2.44) | 1 | 2.40 (2.26-2.55) | 1 |
| Cardiorespiratory fitness | 1.00 | 1.36 (1.27-1.46) | 1 | 1.67 (1.56-1.78) | 2 | 2.27 (2.14-2.40) | 3 | 2.36 (2.22-2.50) | 4 |
| Psychiatric diagnoses | 1.00 | 1.37 (1.28-1.47) | -1 | 1.68 (1.57-1.79) | 0 | 2.26 (2.14-2.40) | 3 | 2.34 (2.20-2.48) | 6 |
| Musculoskeletal diagnoses | 1.00 | 1.36 (1.27-1.46) | 0 | 1.68 (1.57-1.79) | 0 | 2.30 (2.17-2.44) | 1 | 2.41 (2.27-2.55) | 1 |
| Adjusted for all late adolescence variables | 1.00 | 1.29 (1.20-1.39) | 20 | 1.52 (1.42-1.62) | 24 | 1.86 (1.75-1.98) | 34 | 1.81 (1.70-1.93) | 43 |
| Full model | 1.00 | 1.27 (1.18-1.36) | 27 | 1.48 (1.38-1.59) | 29 | 1.81 (1.70-1.93) | 38 | 1.77 (1.66-1.89) | 46 |
| **Long-term unemployment (7 583 events)** | | |  |  |  |  |  |  |  |
| Crude | 1.00 | 1.27 (1.16-1.39) |  | 1.45 (1.33-1.59) |  | 1.80 (1.67-1.94) |  | 1.47 (1.36-1.59) |  |
| *Childhood variables* |  |  |  |  |  |  |  |  |  |
| Parental education | 1.00 | 1.29 (1.18-1.42) | -8 | 1.49 (1.36-1.63) | -8 | 1.87 (1.73-2.02) | -9 | 1.55 (1.42-1.69) | -17 |
| Parental occupation | 1.00 | 1.30 (1.19-1.43) | -11 | 1.50 (1.37-1.64) | -9 | 1.88 (1.74-2.03) | -10 | 1.57 (1.45-1.71) | -22 |
| Crowded housing | 1.00 | 1.26 (1.15-1.38) | 4 | 1.44 (1.31-1.57) | 4 | 1.76 (1.63-1.89) | 5 | 1.43 (1.32-1.55) | 8 |
| Adjusted for all childhood variables | 1.00 | 1.30 (1.19-1.43) | -11 | 1.50 (1.37-1.64) | -10 | 1.87 (1.73-2.03) | -10 | 1.57 (1.44-1.72) | -22 |
| *Late adolescence variables* | |  |  |  |  |  |  |  |  |
| Cognitive ability | 1.00 | 1.23 (1.12-1.35) | 15 | 1.37 (1.25-1.49) | 19 | 1.57 (1.45-1.70) | 28 | 1.22 (1.12-1.33) | 53 |
| Stress resilience | 1.00 | 1.27 (1.16-1.39) | 2 | 1.42 (1.30-1.55) | 7 | 1.68 (1.56-1.81) | 15 | 1.33 (1.23-1.45) | 29 |
| BMI ≥ 25 | 1.00 | 1.27 (1.16-1.39) | 0 | 1.45 (1.33-1.59) | 0 | 1.79 (1.66-1.94) | 0 | 1.47 (1.35-1.59) | 0 |
| Muscle strength | 1.00 | 1.29 (1.18-1.41) | -6 | 1.47 (1.34-1.60) | -4 | 1.82 (1.69-1.96) | -3 | 1.49 (1.38-1.62) | -5 |
| Cardiorespiratory fitness | 1.00 | 1.26 (1.15-1.39) | 3 | 1.43 (1.31-1.56) | 5 | 1.74 (1.61-1.88) | 7 | 1.40 (1.29-1.52) | 14 |
| Psychiatric diagnoses | 1.00 | 1.28 (1.17-1.40) | -2 | 1.45 (1.33-1.58) | 1 | 1.76 (1.63-1.89) | 5 | 1.42 (1.31-1.54) | 11 |
| Musculoskeletal diagnoses | 1.00 | 1.27 (1.16-1.39) | 0 | 1.45 (1.33-1.59) | 0 | 1.80 (1.67-1.94) | 0 | 1.47 (1.35-1.59) | 0 |
| Adjusted for all late adolescence variables | 1.00 | 1.24 (1.13-1.36) | 11 | 1.36 (1.24-1.49) | 20 | 1.52 (1.40-1.65) | 35 | 1.16 (1.06-1.27) | 66 |
| Full model | 1.00 | 1.28 (1.17-1.40) | -3 | 1.41 (1.29-1.55) | 9 | 1.61 (1.48-1.75) | 24 | 1.26 (1.15-1.38) | 45 |

**Supplementary Table 3.** Complete case analysis excluding 18 308 individuals with missing information on potential explanatory factors. Crude and adjusted hazard ratios (HRs) with 95% confidence intervals (CIs) for the association between level of education (in years) and early exit through early old-age retirement with and without income, and percentage of HR reduction (%∆) by each potential explanatory factor.

| Years of education | ≥ 15 | 13-14 | %∆ | 12 | %∆ | 10-11 | %∆ | ≤ 9 | %∆ |
| --- | --- | --- | --- | --- | --- | --- | --- | --- | --- |
| **Early old-age retirement without income (32 860 events)** | | |  |  |  |  |  |  |  |
| Crude | 1.00 | 1.31 (1.26-1.37) |  | 1.44 (1.39-1.50) |  | 1.45 (1.40-1.50) |  | 1.53 (1.47-1.58) |  |
| *Childhood variables* |  |  |  |  |  |  |  |  |  |
| Parental education | 1.00 | 1.30 (1.25-1.36) | 3 | 1.43 (1.37-1.49) | 3 | 1.43 (1.38-1.49) | 3 | 1.51 (1.46-1.57) | 2 |
| Parental occupation | 1.00 | 1.32 (1.26-1.37) | -1 | 1.45 (1.39-1.51) | -1 | 1.46 (1.41-1.51) | -3 | 1.55 (1.49-1.61) | -4 |
| Crowded housing | 1.00 | 1.31 (1.26-1.37) | 0 | 1.44 (1.39-1.50) | 0 | 1.45 (1.40-1.50) | 0 | 1.53 (1.47-1.59) | 0 |
| Adjusted for all childhood variables | 1.00 | 1.31 (1.25-1.36) | 2 | 1.44 (1.38-1.50) | 1 | 1.45 (1.39-1.50) | 0 | 1.53 (1.47-1.59) | -1 |
| *Late adolescence variables* | |  |  |  |  |  |  |  |  |
| Cognitive ability | 1.00 | 1.31 (1.26-1.37) | 0 | 1.44 (1.38-1.50) | 0 | 1.45 (1.39-1.50) | 0 | 1.52 (1.46-1.59) | 1 |
| Stress resilience | 1.00 | 1.31 (1.26-1.37) | 0 | 1.44 (1.40-1.50) | 0 | 1.45 (1.40-1.50) | -1 | 1.53 (1.47-1.59) | -1 |
| BMI ≥ 25 | 1.00 | 1.31 (1.26-1.37) | 0 | 1.44 (1.38-1.50) | 0 | 1.45 (1.40-1.50) | 0 | 1.52 (1.47-1.58) | 0 |
| Muscle strength | 1.00 | 1.31 (1.25-1.36) | 2 | 1.44 (1.38-1.50) | 1 | 1.44 (1.39-1.49) | 1 | 1.52 (1.46-1.57) | 2 |
| Cardiorespiratory fitness | 1.00 | 1.31 (1.26-1.37) | 0 | 1.44 (1.38-1.50) | 0 | 1.44 (1.39-1.49) | 1 | 1.52 (1.46-1.58) | 1 |
| Psychiatric diagnoses | 1.00 | 1.31 (1.26-1.37) | 0 | 1.44 (1.39-1.50) | 0 | 1.45 (1.40-1.50) | 0 | 1.52 (1.47-1.58) | 0 |
| Musculoskeletal diagnoses | 1.00 | 1.31 (1.26-1.37) | 0 | 1.44 (1.39-1.50) | 0 | 1.45 (1.40-1.50) | 0 | 1.53 (1.47-1.58) | 0 |
| Adjusted for all late adolescence variables | 1.00 | 1.31 (1.25-1.36) | 2 | 1.43 (1.38-1.49) | 2 | 1.43 (1.38-1.49) | 4 | 1.50 (1.44-1.56) | 5 |
| Full model | 1.00 | 1.30 (1.25-1.36) | 4 | 1.43 (1.37-1.49) | 3 | 1.43 (1.38-1.49) | 4 | 1.51 (1.44-1.57) | 4 |
| **Early old-age retirement with income (19 634 events)** | | |  |  |  |  |  |  |  |
| Crude | 1.00 | 1.18 (1.12-1.24) |  | 1.12 (1.06-1.18) |  | 1.26 (1.20-1.31) |  | 1.30 (1.24-1.36) |  |
| *Childhood variables* |  |  |  |  |  |  |  |  |  |
| Parental education | 1.00 | 1.18 (1.12-1.24) | 1 | 1.12 (1.06-1.18) | 3 | 1.25 (1.20-1.31) | 2 | 1.29 (1.22-1.35) | 3 |
| Parental occupation | 1.00 | 1.19 (1.13-1.25) | -6 | 1.13 (1.07-1.19) | -7 | 1.27 (1.21-1.33) | -4 | 1.32 (1.26-1.38) | -7 |
| Crowded housing | 1.00 | 1.17 (1.12-1.24) | 2 | 1.11 (1.06-1.17) | 3 | 1.25 (1.20-1.31) | 3 | 1.29 (1.23-1.35) | 3 |
| Adjusted for all childhood variables | 1.00 | 1.18 (1.12-1.25) | -3 | 1.12 (1.06-1.18) | -1 | 1.26 (1.20-1.32) | 0 | 1.30 (1.24-1.37) | -2 |
| *Late adolescence variables* | |  |  |  |  |  |  |  |  |
| Cognitive ability | 1.00 | 1.17 (1.11-1.23) | 6 | 1.10 (1.05-1.16) | 14 | 1.22 (1.17-1.28) | 13 | 1.26 (1.19-1.32) | 14 |
| Stress resilience | 1.00 | 1.18 (1.12-1.24) | -1 | 1.12 (1.07-1.18) | -5 | 1.28 (1.22-1.33) | -7 | 1.32 (1.26-1.38) | -8 |
| BMI ≥ 25 | 1.00 | 1.18 (1.12-1.24) | 0 | 1.12 (1.06-1.18) | 1 | 1.26 (1.20-1.31) | 1 | 1.29 (1.24-1.35) | 1 |
| Muscle strength | 1.00 | 1.17 (1.11-1.23) | 3 | 1.11 (1.06-1.17) | 3 | 1.25 (1.20-1.31) | 2 | 1.29 (1.23-1.35) | 3 |
| Cardiorespiratory fitness | 1.00 | 1.18 (1.12-1.24) | -1 | 1.12 (1.07-1.18) | -3 | 1.27 (1.21-1.32) | -3 | 1.31 (1.25-1.37) | -5 |
| Psychiatric diagnoses | 1.00 | 1.18 (1.12-1.24) | 0 | 1.12 (1.06-1.18) | 0 | 1.26 (1.21-1.32) | -1 | 1.30 (1.24-1.36) | -2 |
| Musculoskeletal diagnoses | 1.00 | 1.18 (1.12-1.24) | 0 | 1.12 (1.06-1.18) | 0 | 1.26 (1.20-1.31) | 0 | 1.30 (1.24-1.36) | 0 |
| Adjusted for all late adolescence variables | 1.00 | 1.16 (1.10-1.22) | 8 | 1.10 (1.05-1.16) | 14 | 1.23 (1.17-1.29) | 10 | 1.27 (1.20-1.33) | 10 |
| Full model | 1.00 | 1.17 (1.11-1.23) | 4 | 1.11 (1.05-1.17) | 10 | 1.24 (1.18-1.30) | 8 | 1.28 (1.21-1.35) | 6 |

**Supplementary Table 4.** Crude and adjusted hazard ratios (HRs) with 95% confidence intervals (CIs) for the association between level of education (years) and each early exit route separately, showing HRs and percentage of HR reduction (%∆) for all conscription variables combined and the full model, as well as all conscription variables combined and the full model except the variable cognitive ability.

| Years of education | ≥ 15 | 13-14 | %∆ | 12 | %∆ | 10-11 | %∆ | ≤ 9 | %∆ |
| --- | --- | --- | --- | --- | --- | --- | --- | --- | --- |
| **Disability pension** |  |  |  |  |  |  |  |  |  |
| Adjusted for all late adolescence variables | 1.00 | 1.33 (1.23-1.44) | 12 | 1.59 (1.47-1.71) | 21 | 2.01 (1.88-2.14) | 35 | 1.95 (1.82-2.08) | 45 |
| All late adolescence variables except cognitive ability | 1.00 | 1.38 (1.28-1.49) | -1 | 1.70 (1.58-1.83) | 6 | 2.30 (2.16-2.45) | 16 | 2.32 (2.18-2.48) | 23 |
| All variables except cognitive ability | 1.00 | 1.35 (1.25-1.45) | 9 | 1.64 (1.52-1.77) | 14 | 2.19 (2.05-2.34) | 23 | 2.22 (2.07-2.38) | 29 |
| Full model | 1.00 | 1.31 (1.21-1.42) | 18 | 1.56 (1.44-1.68) | 25 | 1.96 (1.83-2.10) | 38 | 1.92 (1.79-2.06) | 47 |
| **Long-term sickness absence** | |  |  |  |  |  |  |  |  |
| Adjusted for all late adolescence variables | 1.00 | 1.28 (1.20-1.36) | 17 | 1.51 (1.42-1.61) | 21 | 1.85 (1.75-1.96) | 32 | 1.76 (1.66-1.87) | 41 |
| All late adolescence variables except cognitive ability | 1.00 | 1.32 (1.24-1.41) | 3 | 1.61 (1.52-1.71) | 6 | 2.09 (1.98-2.20) | 13 | 2.05 (1.94-2.17) | 19 |
| All variables except cognitive ability | 1.00 | 1.29 (1.21-1.37) | 13 | 1.56 (1.46-1.65) | 14 | 1.99 (1.89-2.10) | 21 | 1.96 (1.86-2.08) | 25 |
| Full model | 1.00 | 1.26 (1.18-1.34) | 23 | 1.48 (1.39-1.58) | 26 | 1.81 (1.71-1.91) | 35 | 1.74 (1.64-1.84) | 43 |
| **Long-term unemployment** | |  |  |  |  |  |  |  |  |
| Adjusted for all late adolescence variables | 1.00 | 1.27 (1.17-1.38) | 5 | 1.37 (1.26-1.48) | 17 | 1.51 (1.40-1.62) | 33 | 1.16 (1.07-1.25) | 65 |
| All late adolescence variables except cognitive ability | 1.00 | 1.30 (1.20-1.41) | -5 | 1.42 (1.32-1.54) | 5 | 1.64 (1.53-1.76) | 16 | 1.30 (1.21-1.40) | 34 |
| All variables except cognitive ability | 1.00 | 1.33 (1.22-1.44) | -15 | 1.47 (1.35-1.59) | -4 | 1.71 (1.59-1.84) | 6 | 1.39 (1.29-1.51) | 13 |
| Full model | 1.00 | 1.31 (1.20-1.42) | -7 | 1.42 (1.31-1.54) | 7 | 1.59 (1.47-1.71) | 23 | 1.25 (1.16-1.36) | 44 |
| **Early old-age retirement without income** | | |  |  |  |  |  |  |  |
| Adjusted for all late adolescence variables | 1.00 | 1.33 (1.28-1.38) | 3 | 1.46 (1.40-1.51) | 2 | 1.46 (1.41-1.51) | 3 | 1.53 (1.47-1.59) | 3 |
| All late adolescence variables except cognitive ability | 1.00 | 1.33 (1.28-1.38) | 3 | 1.46 (1.40-1.51) | 2 | 1.46 (1.41-1.51) | 3 | 1.53 (1.48-1.59) | 3 |
| All variables except cognitive ability | 1.00 | 1.32 (1.27-1.37) | 5 | 1.45 (1.39-1.51) | 4 | 1.46 (1.41-1.51) | 3 | 1.54 (1.48-1.60) | 2 |
| Full model | 1.00 | 1.32 (1.27-1.37) | 5 | 1.45 (1.39-1.51) | 4 | 1.46 (1.40-1.51) | 4 | 1.53 (1.47-1.59) | 3 |
| **Early old-age retirement with income** | |  |  |  |  |  |  |  |  |
| Adjusted for all late adolescence variables | 1.00 | 1.17 (1.12-1.23) | 9 | 1.11 (1.06-1.17) | 14 | 1.23 (1.18-1.29) | 10 | 1.26 (1.20-1.32) | 9 |
| All late adolescence variables except cognitive ability | 1.00 | 1.18 (1.13-1.24) | 2 | 1.13 (1.08-1.19) | -1 | 1.27 (1.22-1.33) | -5 | 1.31 (1.25-1.37) | -8 |
| All variables except cognitive ability | 1.00 | 1.19 (1.13-1.25) | 1 | 1.13 (1.08-1.19) | -1 | 1.27 (1.21-1.33) | -4 | 1.31 (1.25-1.38) | -10 |
| Full model | 1.00 | 1.18 (1.12-1.24) | 6 | 1.12 (1.06-1.18) | 11 | 1.24 (1.18-1.29) | 9 | 1.27 (1.21-1.34) | 5 |
